# Ki-67 expression predicts locoregional recurrence in stage I oral tongue carcinoma

**DOI:** 10.1038/sj.bjc.6604633

**Published:** 2008-09-02

**Authors:** D Wangsa, M Ryott, E Åvall-Lundqvist, F Petersson, G Elmberger, J Luo, T Ried, G Auer, E Munck-Wikland

**Affiliations:** 1 Department of Oncology-Pathology, Karolinska Institute, Karolinska University Hospital, Stockholm, Sweden; 2 Genetics Branch, Center for Cancer Research, National Cancer Institute, National Institutes of Health, Bethesda, MD, USA; 3 Department of Oto-Rhino-Laryngology, Head and Neck Surgery, Karolinska University Hospital, Stockholm, Sweden; 4 Department of Gynaecologic Oncology, Radiumhemmet, Karolinska University Hospital, Stockholm, Sweden; 5 Department of Medical Epidemiology and Biostatistics, Karolinska Institutet, Stockholm, Sweden

**Keywords:** oral cancer, Ki-67, locoregional recurrence, ploidy, genomic instability

## Abstract

Oral tongue squamous cell carcinoma (OTSCC) is an aggressive cancer associated with poor prognosis. Methods for determining the aggressiveness of OTSCC from analysis of the primary tumour specimen are thus highly desirable. We investigated whether genomic instability and proliferative activity (by means of Ki-67 activity) could be of clinical use for prediction of locoregional recurrence in 76 pretreatment OTSCC paraffin samples (stage I, *n*=22; stage II, *n*=33; stage III, *n*=8; stage IV, *n*=13). Eleven surgical tumour specimens were also analysed for remnants of proliferative activity after preoperative radiotherapy. Ninety-seven percent of cases (*n*=72) were characterised as being aneuploid as measured by means of image cytometry. Preoperative radiotherapy (50–68 Gy) resulted in significant reduction of proliferative activity in all patients for which post-treatment biopsies were available (*P*-value=0.001). Proliferative activity was not associated with response to radiation in stage II patients. However, we report a significant correlation between high proliferation rates and locoregional recurrences in stage I OTSCC patients (*P*-value=0.028). High-proliferative activity is thus related to an elevated risk of recurrence after surgery alone. We therefore conclude that Ki-67 expression level is a potentially useful clinical marker for predicting recurrence in surgically treated stage I OTSCC.

Oral tongue squamous cell carcinoma (OTSCC) is an aggressive cancer frequently associated with poor prognosis. Five-year survival rates remained essentially unchanged over the past 20 years despite advancements in treatment (Myers *et al*, 2000; Forastiere *et al*, 2001; Annertz *et al*, 2002; Brenner, 2002; Shiboski *et al*, 2005). This is partly due to patients dying from metastatic disease despite being diagnosed at an early stage (Sano and Myers, 2007). Detection of occult metastases is difficult, which is why prognostic markers in primary diagnostic tumour specimens are highly desirable.

Treatment failure in OTSCC patients is most frequently due to local and regional recurrences, with the most important prognostic indicator being the presence of metastasis in cervical lymph nodes (Kalnins *et al*, 1977; Alvi and Johnson, 1996; Ferlito *et al*, 2001; Sano and Myers, 2007). Early stage OTSCC patients generally have a 2-year survival rate of more than 85%, although the survival rate decreases by approximately 50% with the finding of cervical nodal metastasis (Sano and Myers, 2007). Some studies have shown a high rate of occult nodal metastasis (20–40%) despite any evidence of regional spread on clinical or radiographic evaluation (Yuen *et al*, 1999; Ferlito and Rinaldo, 2000). The high rate of occult metastases is attributable to technological limitations (Lydiatt *et al*, 1993; Alvi and Johnson, 1996; Kademani, 2007; Bilde *et al*, 2008). As lymph node involvement occurs independent of tumour size, small primary tumours may have cervical lymph node metastasis whereas larger tumours do not (Sano and Myers, 2007). Predictive markers indicating a high risk for lymph node metastasis would have a significant role in determining the therapeutic strategy for these patients (Sano and Myers, 2007).

Genomic instability and proliferative activity are important factors for tumour progression and metastatic growth in cancers. Several studies have shown that aneuploid tumours are more aggressive than diploid tumours in head and neck cancers (Hogmo *et al*, 1994; Rubio Bueno *et al*, 1998). Proliferative activity, as determined by expression levels of the Ki-67 nuclear antigen, has been linked to prognosis and treatment prediction with varying results in oral cancer, with few studies performed exclusively in OTSCC (Xie *et al*, 1999; Pich *et al*, 2004; Lothaire *et al*, 2006; Kim *et al*, 2007). The use of immunohistochemistry and DNA cytometry are cost-effective and robust clinical tests. We hereby investigate whether Ki-67 expression and ploidy measurements can be of clinical use for prediction of locoregional recurrence exclusively in primary OTSCC.

## Materials and methods

### Patients

Seventy-six formalin-fixed, paraffin-embedded pretreatment biopsy specimens with histopathologically confirmed OTSCC, UICC stages I–IV, treated at the Department of Oto-Rhino-Laryngology, Karolinska University Hospital (Stockholm, Sweden) from January 2000 to December 2004 were investigated. Eleven surgical specimens were obtained from stage II patients following preoperative radiation, in addition to the biopsy specimens already collected. All histological samples were reviewed by an experienced pathologist (GE) who was blinded to clinical outcome. Clinical information, including age, tumour grading, treatment modality, treatment response and follow-up according to stage were retrieved from the medical records. The present study was carried out with approval from the Research Ethical Review Board in Stockholm.

**Figure 1:**
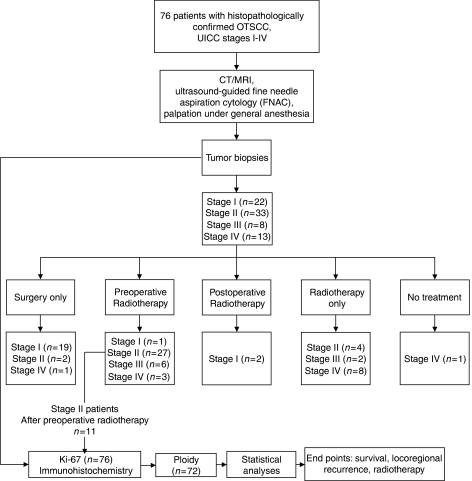
Pictorial presentation of specimen accrual, treatment and experimental design. UICC refers to International Union Again Cancer.

The general treatment for OTSCC depended on the UICC classification stage and patient performance status (Greene *et al*, 2002). The stage and size of the tumour was determined prior to treatment plan with CT/MRI, ultrasound-guided fine-needle aspiration cytology and palpation under general anaesthesia. A specific treatment plan for each patient was decided at a conference between head and neck surgeons, oncologists and pathologists. Standard treatment at Karolinska University Hospital during the time of study for stage I OTSCC consisted of local resection alone. Standard treatment for stage II OTSCC included preoperative radiotherapy against the tumour and ipsilateral neck nodes followed by hemiglossectomy 4–6 weeks after radiotherapy treatment was concluded. Deviations in treatment could occur depending on the growth pattern and radiation response of the tumour. Patients diagnosed with OTSCC stages III and IV were treated individually according to their size and spread, with surgery and/or radiotherapy and/or chemotherapy. Generally preoperative radiotherapy was given with a total dose of 50–68 Gy, which was a normal preoperative radiotherapy dose for head and neck cancers in Stockholm. The radiation range varied depending on the clinical presentation of the patient and growth pattern of the tumour. After preoperative radiotherapy, surgery was performed as soon as possible to avoid delay time. The time between preoperative radiotherapy and surgery, usually 4–6 weeks, depended on the patient's recovery time for optimal surgical treatment. Pictorial presentation of treatment and experimental design is shown in Figure 1.

Evaluation of radiation response was performed by histopathological examination of the formalin-fixed surgical specimen and was classified as complete pathological remission (pCR) if no morphological intact tumour cells were found. Incomplete pathological remission (non-pCR) was classified when remnant tumour cells were detected histopathologically.

### Ki-67 immunohistochemical staining

Slides used for immunohistochemical staining were cut into 4 *μ*m sections. Two 4 *μ*m sections were cut before and after the immunohistochemical sections for haematoxylin-eosin (HTX) staining. The HTX slides were evaluated by our pathologist (FP) to confirm tumour representativity for the Ki-67 slides. Immunohistochemistry of molecular markers for Ki-67 was performed a week after cutting, using the Benchmark XT system, a product of Ventana Medical Systems, which automatically prepares and stains the 4 *μ*m Ki-67 slide sections. All 76 slides were placed in the machine at the same time to avoid any discrepancies in staining. We used the monoclonal antibody MIB-1 (DAKO, Glostrup, Denmark). Immunohistochemistry of Ki-67 was performed on 76 tumour tissue sections. Tonsil cancer served as a positive control for the staining. The Benchmark XT system has an external-negative control to the primary antibody during each run. Staining reproducibility was verified by staining five new slides of previously stained Ki-67 cases for comparison purposes. The results, according to our pathologist (JL), showed that the slides were similarly stained and produced the same score as the previous batch. With standardised staining/analysis techniques, the results were thus reproducible.

### Ki-67 immunohistochemical evaluation

The stained Ki-67 slides were analysed according to their nuclear staining pattern using the VIAS workstation, a Ventana Image Analysis System. Using the combined automatic segmentation tool, four representative tumour areas on the sections were manually chosen on low power ( × 40) magnification and automatic counting was performed at × 400 magnification ( × 40 objective). One pathologist (FP) performed the analysis.

Approximately 1000 cells were evaluated per case, with the given percentages from 0 to 100, with 0 being no nuclear staining to 100 being total nuclear staining of the cells. The analyses were performed without prior knowledge of the clinical outcome. Ki-67 expression comparisons were made by using the percentages (0–100%) as a continuous variable and by dividing the scores into two categorical groups. The two categorical groups consisted of two different cutoff points, with the first at 50 (low: 0–50, high: 51–100), and the second at 32 (low: 0–32, high: 33–100). The first cutoff value at 50 was chosen as being half the value of the continuous variable. The second cutoff value at 32 was chosen for statistical purposes according to a similar study by Davies *et al* (2006).

### DNA cytometry

Image cytometry was to be performed on 76 pretreatment biopsy sections (8 *μ*m) with tumour representativity confirmed by our pathologist (GE) with corresponding HTX slides. Four patient samples were eliminated due to tumour non-representativity. Of 72, 8 *μ*m slides were then Feulgen stained to measure the nuclear DNA content of the tumour cells. The staining, internal standardisation, and tumour cell selection were based on previously described methods (Steinbeck *et al*, 1999). DNA values were determined in relation to a corresponding control, which denoted the normal DNA (diploid) content at 2c. The specimens were divided into two groups by their corresponding histogram. Histograms with stem lines in the 2c region and no cells exceeding 4c represented diploid tumours. Aneuploid tumours were denoted by one or more peaks outside the 2c region and a substantial number of cells with DNA values exceeding the 4c region. Approximately 100 cells were analysed for each tumour specimen.

### Statistical analysis

The data was statistically analysed by means of the SAS version 9.1 software (SAS Institute: Cary, NC, USA). The Mantel–Haenszel *χ*^2^-test (*χ*^2^ MH) was used to assess associations between Ki-67 categorical variables and clinicopathologic factors. The Kruskal–Wallis test was used for continuous variable assessments between clinicopathologic factors and the immunohistochemical biomarker. The log-rank test was used to screen for clinical and immunohistochemical prognostic values. The Kaplan–Meier survival curves were constructed to compare differences in survival, using a 60-month cutoff with the Stata/IC10 software. Survival was calculated from the date of tumour diagnosis until the time of death from any cause, or in patients who remained alive, the time of last follow-up (follow-up time varied from 36 to 60 months). All *P*-values were from a two-sided test with a *P*-value <0.05 considered to indicate statistical significance.

## Results

### Clinicopathologic and immunohistochemical analyses

Baseline patient characteristics were reviewed according to cancer stages I–IV (Table 1). Fifty percent of the patients were dead following a minimum 3-year follow-up since cancer diagnosis, with a median survival time of 22 months (mean: 27 months, range: 3–60 months). In early OTSCC, 23 out of 55 patients in stages I and II had a recurrence. Out of which, 22 patients had locoregional recurrences (4 local recurrences and 18 regional recurrences) and 1 patient had a systemic recurrence. In stage I OTSCC, one patient received post-operative radiotherapy.

**Figure 2:**
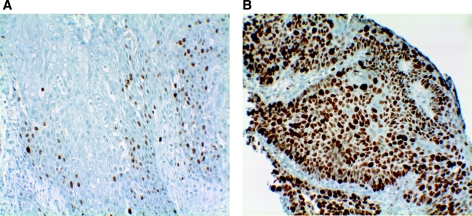
Immunohistochemical detection in primary pretreatment biopsies of: (**A**) a low percentage (17%) of Ki-67-positive nuclear staining; (**B**) a high percentage (93%) of Ki-67-positive nuclear staining.

Ki-67 nuclear staining could be detected in all 76 formalin-fixed biopsy specimens and 11 formalin-fixed surgical tumour samples analysed. Ki-67 expression did not correlate to stage (*P*-value=0.181). In addition, statistical analyses were made between Ki-67, DNA content, and resection status, with no correlation seen between the three parameters. The percentage of Ki-67-positive cells within a tumour sample ranged from 17 to 95% with a median of 56%. Figure 2A illustrates a low-proliferative tumour and Figure 2B demonstrates a high-proliferative tumour. The first Ki-67 categorical division contained 29 samples in the low percentage group (0–50) and 47 samples in the higher Ki-67 percentage group (51–100). The second Ki-67 categorical division contained 10 samples in the low percentage group (0–32), and 66 samples in the higher percentage group (33–100).

### DNA cytometry analyses

Image cytometric measurements on pretreatment biopsies resulted in 97% aneuploid and 3% diploid tumours (*n*=72). Two stage I tumours characterised as diploid were alive after follow-up time ended. One patient with a diploid tumour had no recurrence whereas the second patient had a secondary primary tumour (Ki-67-positive cells: 49 and 32%, respectively). The fact that the majority of cancers were aneuploid, prevented a meaningful statistical analysis of a potential association between genomic instability and recurrence.

### Prediction of locoregional recurrences

Ki-67 levels were not associated with locoregional recurrence in stages II–IV tumours. However, a significant correlation (*P*-value=0.028) was detected between Ki-67 expression in stage I primary tumours and locoregional recurrences. A higher Ki-67 expression in stage I correlated with a higher number of locoregional recurrences in two of three assessments (Table 2). After adjusting for stage and recurrence in a multivariate analysis, a significant correlation was not detected in Ki-67 levels.

### Ki-67 response to radiotherapy

In stage II OTSCC, 27 out of 33 patients underwent preoperative radiotherapy. Radiotherapy response was measured pathologically with 7 out of 27 pCR and 20 out of 27 non-pCRs. We could not detect any significant differences in Ki-67 expression between pCR and non-pCR cases.

Ki-67 expression levels were evaluated before and after radiotherapy treatment in eleven stage II patients. These patients were classified as non-responders to radiotherapy. Ki-67 levels decreased in all eleven patients in comparison to their pretreatment specimens (Table 3).

### Survival outcome

**Figure 3:**
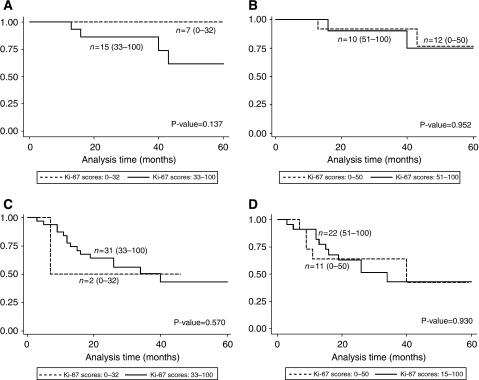
Kaplan–Meier survival estimates in relation to (**A**) Ki-67-expression variables (0–32, 33–100) in stage I oral tongue squamous cell carcinoma (OTSCC); (**B**) Ki-67-expression variables (0–50, 51–100) in stage I OTSCC; (**C**) Ki-67-expression variables (0–32, 33–100) in stage II OTSCC; (**D**) Ki-67-expression variables (0–50, 51–100) in stage II OTSCC.

The relationship between Ki-67 expression and overall survival was assessed in stages I–IV. We did not observe any statistical differences in overall survival between Ki-67 expression and tumour stage. However, in stage I OTSCC a trend is observed, with patients exhibiting low Ki-67 expression (Ki-67⩽32%) tending to fare better than patients with Ki-67 expression above 32% (Figure 3A). This trend was not observed in stage II OTSCC for the same Ki-67 categorical group (0–32 and 33–100), shown in Figure 3C. In the Ki-67 categorical groups (0–50, 51–100), differences in overall survival were not detected for either stages I or II OTSCC (Figure 3B and D).

In stage II, patients treated with preoperative radiotherapy, three of seven pCR patients died with two of the three patients having locoregional recurrences. In the non-pCR group, 8 of 20 patients died with 7 showing locoregional recurrences. All patients who had locoregional recurrences died within approximately 3 years.

## Discussion

To identify patients with a high risk for locoregional recurrence, we investigated Ki-67 expression as a possible marker in OTSCC. We found an association between high-proliferative activity and an increased incidence of locoregional recurrences in stage I OTSCC. We did not observe a relationship between proliferative activity and radiotherapeutic response; however, we did detect a decreased Ki-67 expression in residual post-radiotherapy stage II tumours. In addition, we detected a survival trend in stage I tumours, although not significant, where patients with a proliferation score below 33 tended to fare better.

The degree of aneuploidy is an independent predictor of clinical outcome. Studies have shown that patients with highly aneuploid tumours have reduced disease-free survival times compared to patients with less unstable tumours (ie breast and colorectal cancer; Armitage *et al*, 1985; Hemmer *et al*, 1990; Kronenwett *et al*, 2006). In oral cancers, ploidy studies have yielded varying degrees of aneuploidy from 50 up to 70% (Hemmer *et al*, 1990; Baretton *et al*, 1995). Our study represented a large aneuploid OTSCC population (97%), which reflects high genomic instability in our material. The differences in ploidy results could be due to the inclusion of the entire oral cavity for most studies whereas our study encompasses only OTSCC material.

High-proliferative activity (>50%) is related to an elevated recurrence risk after surgery in patients with stage I tumours, making Ki-67 a potentially useful marker for patients in need of more extensive treatment (ie surgery with more extensive margins, neck dissection and postoperative radiotherapy). The high rate of metastasis in stages I and II tumours is in accordance with previous studies that show a failure rate at 20–40% (Sano and Myers, 2007). Earlier studies on Ki-67 expression in locoregional recurring oral cancers revealed conflicting results (Grabenbauer *et al*, 2000; Koelbl *et al*, 2001; Lavertu *et al*, 2001; Davies *et al*, 2006; Wilson *et al*, 2006). Two studies have suggested that a Ki-67 labelling index of more than 20% was associated with a significantly worse locoregional control (56%) in oropharyngeal cancer (Grabenbauer *et al*, 2000; Wilson *et al*, 2006). This is in agreement with our results that found that high-proliferative activity is associated with an elevated risk for recurrence. The study by Davies *et al* (2006), however, showed that a 0–33% Ki-67 expression was associated with a six-times-greater risk of recurrence at the leading edge of early stage OTSCC. These apparent contradictory results could be due to Davis collecting all node-negative OTSCC, which incorporates both stages I and II tumours. Our findings investigated the relationship between Ki-67 expression and tumours from only stage I OTSCC. In addition, we used an automatic staining and counting method that decreases variability and increases objectivity in the analysis of the Ki-67 expression.

Today we lack the means of predicting radiosensitivity outcome in OTSCC patients, with the variability of radiotherapy response in patients with tongue cancer disturbingly high. In our study, we observed a wider range of radiation dosages (50–64 Gy), which was determined according to specific tumour growth and radiation response. The wider range of radiation dosage may have an effect on why many patients in our study did not completely respond to radiotherapy. Patients treated with preoperative radiotherapy experienced complete pathological remission (8 of 36) although the majority (28 of 36) responded only incompletely. We were unable to determine whether the specific time between preoperative radiotherapy and surgery would influence the patient. However, assuming that any delay in surgery would be harmful to the patient, preoperative radiotherapy is therefore an unfavourable treatment for the patient. Two stage II patients that completely responded to radiotherapy and surgery presented with locoregional recurrences a year and a half after diagnosis, making it possible that micrometastases were present. The knowledge of micrometastases would have encouraged further treatment.

Several studies have provided evidence that with a higher proliferative activity, head and neck cancers may respond significantly better to radiotherapy (Raybaud-Diogene *et al*, 1997; Grabenbauer *et al*, 1998; Kropveld *et al*, 1998; Couture *et al*, 2002). Evidence has shown that Ki-67 may be a potential marker for radiosensitivity in head and neck carcinomas when using a cutoff point at <20 or ⩾20% (Raybaud-Diogene *et al*, 1997; Grabenbauer *et al*, 1998; Couture *et al*, 2002). Koelbl *et al* (2001), however, did not find a correlation between Ki-67 expression and radiation response, but an improved local control in tumours with complete response after radiotherapy (40 Gy). The consistently lower proliferation rate found in resected sections after preoperative radiotherapy as compared to the diagnostic specimen in all patients could perhaps signify radiotherapy treatment response in the patient. The varying degrees of change observed before and after radiotherapy indicate that the individual response to radiotherapy varies and most certainly depends on many different factors and is difficult to predict. Our study, which was performed exclusively on OTSCC, corroborated this interpretation: we found no evidence that Ki-67 is a useful marker for predicting radiosensitivity in OTSCC patients.

When assessing overall survival in OTSCC patients, it is important to consider the impact of tumour stage. Previous studies in many different types of cancers have shown that tumour size and patient survival are correlated (Sargeran *et al*, 2007; Kim *et al*, 2008). In this study, we therefore examined whether Ki-67 expression by stage impacts overall survival in OTSCC. Our results indicated that, although not significant, stage I patients with a lower percentage (0–32%) of Ki-67 positively stained cells had a better overall survival. Ki-67 expression did not correlate with overall survival in any other stage, possibly because Ki-67 expression below 33% was generally observed in only stage I OTSCC. Other studies in oral cancer investigating the correlation between Ki-67 expression and overall survival in patients found conflicting results although several studies confirmed that a lower Ki-67 expression exhibited better patient survival (Gonzalez-Moles *et al*, 1996; Piffko *et al*, 1996; Stoll *et al*, 2000; Myoung *et al*, 2006; Kim *et al*, 2007). One study by Kim *et al* (2007) observed a significantly longer patient survival when Ki-67 expression was below 10% in OTSCC patients. As we did not have many OTSCC patients with a Ki-67 expression below 10%, we were unable to corroborate the significance in overall survival at this threshold. In addition, most studies investigating the correlation between Ki-67 expression and patient overall survival examined the entire oral cavity and not OTSCC as a separate entity, which complicates direct comparison. A more favourable prognosis has been seen in studies assessing overall survival in patients with complete pathologically response after radiotherapy, although this was not seen in our study (Brun *et al*, 2001; Onizawa *et al*, 2006).

Most studies investigating the clinical value of markers include tumours from all sub-sites of head and neck cancers. As for instance, tonsillar cancer and OTSCC show large biological differences, in terms of radiosensitivity and prognosis, we advocate the importance of investigating the sub-sites separately. Even base of tongue cancer and OTSCC cases ought to be separated because base of tongue cancer show more similarities to tonsillar cancer than OTSCC, ie HPV positivity (Dahlgren *et al*, 2004). This is our reason for focusing specifically on OTSCC.

In stage I OTSCC, a correlation between Ki-67 expression and survival was seen in scores below 33, however that correlation was not seen once the categorical cutoff was at 0–50. In comparison, a correlation between Ki-67 expression and locoregional recurrence was seen in the higher categorical cutoff (0–50 *vs* 51–100) but not in the lower categorical cutoff (0–32 *vs* 33–100). Due to inconsistent findings in previous publications, correlations between Ki-67 expression and patient characteristics such as survival and locoregional recurrence remain controversial. However, although our analysis in stage I OTSCC is based only on 22 cases, by specifically looking at a specific stage, we attempted to eliminate any confounding factors that stage may have on Ki-67. In addition, as our tissue was specific for only OTSCC, we believe this would eliminate any tissue-specific differences during interpretation.

In this study, Ki-67 expression was examined by immunohistochemistry. Due to the high cost for treatment in oral cancer, the discovery of a molecular marker using immunohistochemistry would be the most cost-effective means for treatment selection (Menzin *et al*, 2007). This study was limited by our small population size; however, OTSCC is a sub-site specific cancer with very few published articles. Methodologically, different approaches in Ki-67 immunohistochemical evaluation were used, making comparison difficult due to lack of one standardised assessment method (Pich *et al*, 2004; Kim *et al*, 2007). In addition, a limitation of the study is the heterogeneity of treatment modalities among the different stages of OTSCC. In this study, a positive methodological aspect is the use of the Ventana machines to stain and count the tumour specimen. This allowed for a more objective quantification of the Ki-67 immunohistochemical marker. In addition, each slide was stained with the same antibody batch and evaluated in one sitting, making comparisons between slides more accurate. Despite the many negative reports in the literature, proliferative activity measurement may still be a reliable prognostic factor when studies are performed with well-standardised methodology.

In summary, we found that OTSCC are genomically unstable cancers. We found that high Ki-67 expression is correlated with locoregional recurrence in stage I OTSCC patients, making it a potential marker for additional therapy in patients. However, additional studies in a larger cohort of patients are warranted before Ki-67 can be used in clinical setting.

## Figures and Tables

**Table 1:** Patient and tumour characteristics according to stage

**Characteristics**	**Stage I^a^ *n*=22**	**Stage II^a^ *n*=33**	**Stage III^a^ *n*=8**	**Stage IV^a^ *n*=13**	**Total *n*=76 (%)**
*Age at diagnosis (years)*					
20–39	3	5	1	1	10 (13)
40–59	10	15	4	2	31 (41)
>59	9	13	3	10	35 (46)
					
*Gender*					
Male	9	23	5	7	44 (58)
Female	13	10	3	6	32 (42)
Smoking habit					
Smoking	11	17	5	6	39 (51)
Non-smoker	7	12	2	2	23 (30)
No information	4	4	1	5	14 (18)
					
*Histopathologic grade* b					
Well differentiated	9	5	1	3	18 (24)
Moderately differentiated	11	22	7	7	47 (62)
Poorly differentiated	2	6	0	3	11 (14)
					
*Preoperative radiation response*					
pCR	0	7	1	0	8 (11)
Non-pCR	0	20	5	3	28 (37)
					
*Survival*					
Alive	17	16	2	3	38 (50)
Dead	5	17	6	10	38 (50)
					
*Resection status*					
R0	22	27	5	4	58 (76)
R1	0	1	0	1	2 (3)
R2	0	0	0	0	0 (0)
No primary surgery	0	5	3	8	16 (21)
					
*Recurrence*					
Locoregional	9	13	NA	NA	NA
Systemic	0	1			
No recurrence	11	19			
Secondary primary	2	0			
					
*Ki-67*					
Mean	48	59	56	60	NA
Range	17–80	18–95	33–87	19–93	

Abbreviations: pCR, complete pathological remission; NA, not applicable; non-pCR, incomplete pathological remission; R0, no gross residual disease and negative margins of resection; R1, residual microscopic disease; R2, residual gross disease.

a TNM stage, tumour stage according to UICC.

b Tumour differentiation grade according to WHO international histologic classification of tumours.

**Table 2:** KI-67 expression in relation to locoregional recurrences of early oral tongue squamous cell carcinoma stages

**Stages**	**Markers**	**Variables**	**Local/regional recurrence**	**No recurrence**	***P*-value**
1 (*n*=22)	Ki-67	33–100	8/9 (89%)	6/11 (55%)	*P*=0.104^a^
		0–32	1/9 (11%)	5/11 (45%)	
					
	Ki-67	51–100	7/9 (78%)	3/11 (27%)	*P*=0.028^a^
		0–50	2/9 (22%)	8/11 (73%)	
					
	Ki-67	Range	32–80	17–71	
	*(continuous)*	Mean/average	59	40	*P*=0.030^a^
		Median	58	41	
2 (*n*=33)	Ki-67	33–100	13/13 (100%)	17/19 (89%)	*P*=0.265^a^
		0–32	0/13 (0%)	2/19 (11%)	
					
	Ki-67	51–100	10/13 (77%)	11/19 (58%)	*P*=0.394^a^
		0–50	3/13 (23%)	8/19 (42%)	
					
	Ki-67	Range	43–95	18–93	
	*(continuous)*	Mean/average	62	56	*P*=0.459^a^
		Median	56.5	59	

a *χ*^2^ MH test.

**Table 3:** KI-67 protein expression in pretreatment and post-treatment biopsies in non-pCR stage 2 OTSCC

**Age^a^**	**Sex**	**Pretreatment biopsy (%)**	**Post-treatment biopsy^b^ (%)**	**Δ change**	**Recurrence**	**Survival**
58	F	58	27	31	None	Alive
33	M	81	18	63	Locoregional	Dead
38	F	53	24	29	None	Alive
54	F	50	41	9	Locoregional	Dead
53	M	43	31	12	Locoregional	Dead
32	F	68	60	8	None	Alive
58	M	49	35	14	Locoregional	Alive
61	M	55	54	1	Locoregional	Dead
21	M	58	47	11	Locoregional	Dead
57	M	66	28	38	None	Alive
75	F	72	53	19	None	Alive
*P*-value				*P*=0.001		
Mean		59	38	21	NA	NA
s.d.		11.3	13.9	17.8		

a Patients received preoperative radiation (50–68 Gy).

b Non-pCR (incomplete pathological remission).
